# Itiner-e: A high-resolution dataset of roads of the Roman Empire

**DOI:** 10.1038/s41597-025-06140-z

**Published:** 2025-11-06

**Authors:** Pau de Soto, Adam Pažout, Tom Brughmans, Peter Bjerregaard Vahlstrup, Álvaro Auir, Toon Bongers, Jens Emil Bødstrup Christoffersen, Maël Crépy, Mathias Holland Johansen, Joseph Lewis, Louis Manière, Michele Rüzgar Massa, Louise Matilde Harreby Møller, Bérangère Redon, Giuseppina Renda, Hamdi Şahin, Adéla Sobotková, Amanda Leighton Spatzek, Philip Verhagen, Barbora Weissova

**Affiliations:** 1 Grup de Recerca en Arqueologia (GRAC_UAB), Universitat Autònoma de Barcelona, Barcelona, Catalonia Spain; 2 Department of History and Classical Studies, Social Resilience Lab, and Centre for Urban Network Evolutions (UrbNet), Aarhus University, Aarhus, Denmark; 3 Center for Humanities Computing, Aarhus University, Aarhus, Denmark; 4 University of Barcelona, Barcelona, Spain; 5 Department of History, Ghent University Ghent, Belgium; 6 Aarhus University, Aarhus, Denmark; 7 French National Centre for Scientific Research, HiSoMA Research Center (UMR 5189), Lyon, France; 8 Gothenburg university, Department of history, Gothenburg, Sweden; 9 Department of Archaeology, University of Cambridge, Cambridge, United Kingdom; 10 Bilkent University, Ankara, Turkey; 11 Department of Humanities and Cultural Heritage, University of Campania Luigi Vanvitelli, Santa Maria Capua Vetere, Italy; 12 Istanbul University, Istanbul, Turkey; 13 Department of History and Classical Studies, Aarhus University, Aarhus, Denmark; 14 Faculty of Social Sciences and Humanities, Vrije Universiteit Amsterdam Amsterdam, Netherlands; 15 Institute of Classical Archaeology, Faculty of Arts, Charles University Prague, Czechia

**Keywords:** Archaeology, History

## Abstract

The Roman Empire’s road system was critical for structuring the movement of people, goods and ideas, and sustaining imperial control. Yet, it remains incompletely mapped and poorly integrated across sources despite centuries of research. We present Itiner-e, the most detailed and comprehensive open digital dataset of roads in the entire Roman Empire. It was created by identifying roads from archaeological and historical sources, locating them using modern and historical topographic maps and remote sensing, and digitising them with road segment-level metadata and certainty categories. The dataset nearly doubles the known length of Roman roads through increased coverage and spatial precision, and reveals that the location of only 2.737% are known with certainty. This resource is transformative for understanding how mobility shaped connectivity, administration, and even disease transmission in the ancient world, and for studies of the millennia-long development of terrestrial mobility in the region.

## Background & Summary

The study of roads of the Roman Empire is a centuries-old pursuit^1,2^. There is a wealth of information about roads that were physically identified in archaeological excavations and surveys, about milestones which were placed at regular intervals along Roman roads, and historical sources like the *Antonine Itinerary*^3,4^ or the *Tabula Peutingeriana*^5^, describing major connections between settlements, as well as detailed regional summaries on Roman roads^6,7^. However, finding and locating this diversity of research and the spatially precise locations of the roads themselves is inhibited by a lack of an Empire-wide synthesis and digitisation.

There have previously been very few other open digital representations of our knowledge of roads over the entire Roman Empire. The *Barrington Atlas of the Greek and Roman World*^8^ is the main reference atlas for the ancient world, and the roads recorded in it are openly digitally available from the Ancient World Mapping Center (https://awmc.unc.edu) and Mapping Past Societies (formerly the Digital Atlas of Roman and Medieval Civilizations, https://darmc.harvard.edu/). These two digitisations are highly similar and differ only in a few areas where the latter also includes roads from the *Tabula Imperii Byzantini* for Greece and Asia Minor^9–16^. They have proven invaluable resources for spatially explicit computational studies of ancient trade and mobility, and as references for the overall structure of the Roman transport system^17,18^. However, both are of limited spatial detail as they reflect the spatial resolution of the *Barrington Atlas* (ranging from 1:500,000 to 1:1,000,000), which results in disregarding natural corridors and barriers. Moreover, the sources used to create the roads included in the *Barrington Atlas* are only available as a short list of references for all information in an atlas map sheet, and not on a road-by-road basis. The sources and methods used to include roads in the *Barrington Atlas* and trace their particular paths are not documented, and a quantitative assessment of how representative and reliable this resource is is not possible.

Itiner-e (dataset: 10.5281/zenodo.17122148, online platform: https://itiner-e.org/) addresses this gap in resources for studying the ancient world. It presents an open, spatially high resolution digitisation of Roman roads, drawing on published historical and archaeological information, topographic maps, and remote sensing data, citing the sources used to create each road segment, and making each road segment uniquely citable through a URI (Uniform Resource Identifier) that is linked to URIs of nearby ancient places in the Pleiades gazetteer of ancient places (http://pleiades.stoa.org/). Itiner-e covers the area of the Roman Empire at its maximum extent, ca. 150 CE, and includes any terrestrial route with an evidenced, conjectured, or hypothesised location in the sources used for the study. This includes any road predating Roman conquest that continued to be in use during Roman times.

**Fig. 1:**
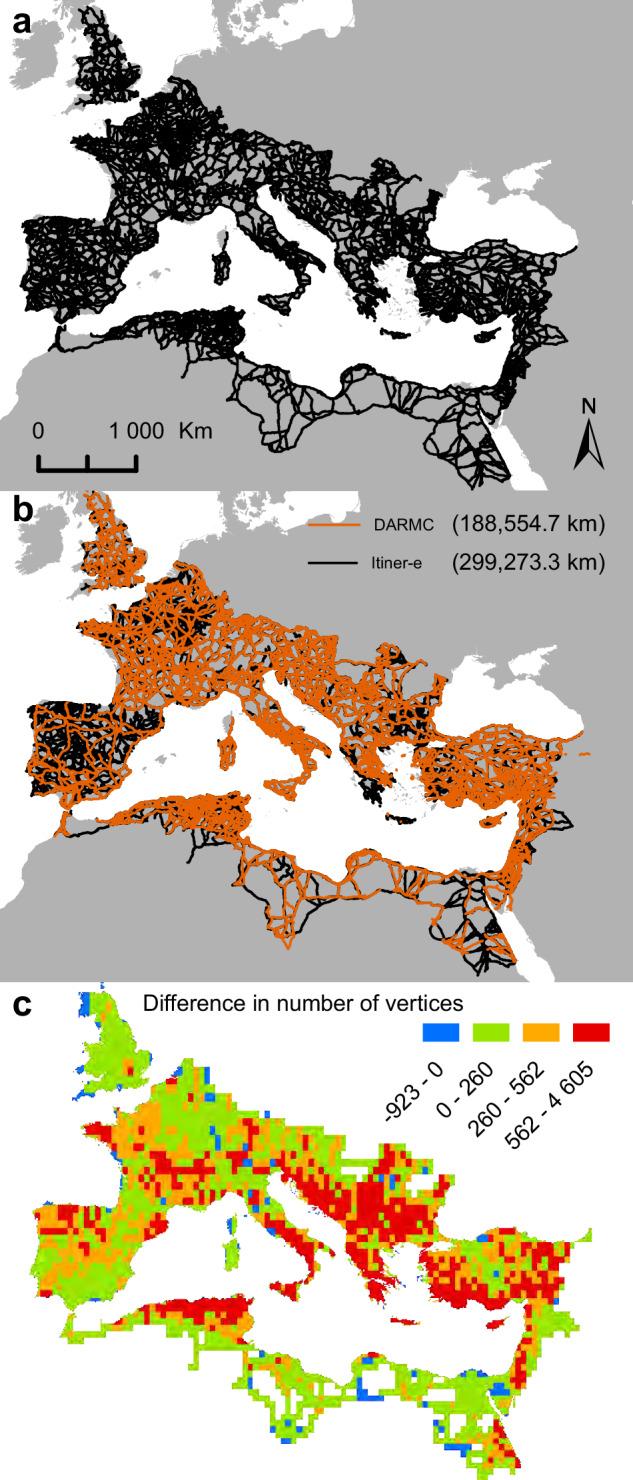
(**a**) The Itiner-e road dataset, and two comparisons with the DARMC dataset: (**b**) difference in coverage, and (**c**) difference in spatial resolution as expressed by the number of vertices contained in the datasets. Itiner-e increases the coverage in selected areas throughout the Empire, most notably in the Iberian Peninsula, the Aegean and Egypt, and increases spatial detail throughout with a few exceptions marked in blue in c).

**Fig. 2:**
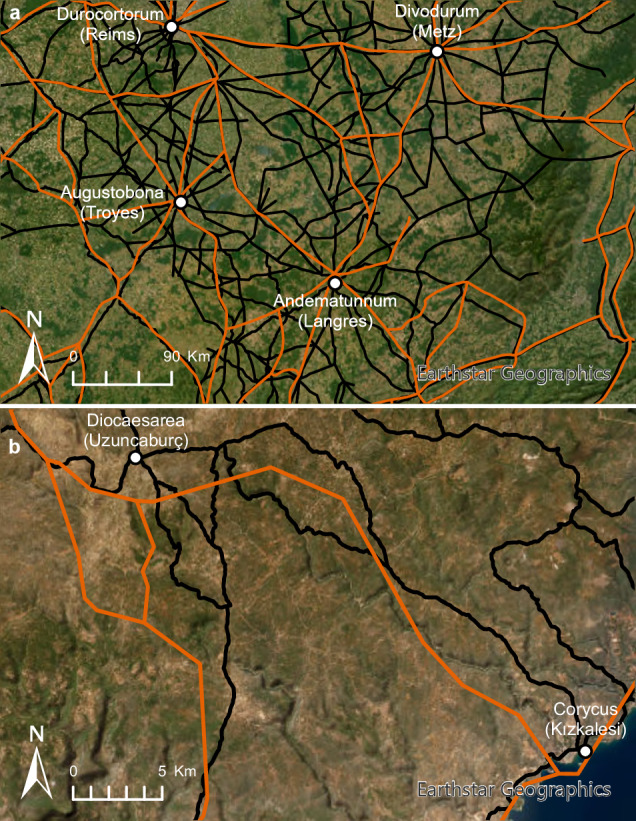
Comparison of DARMC (in orange) and Itiner-e (in black) datasets, (**a**) showing an example from France of a region with increased coverage of roads, and (**b**) an example of increased spatial detail.

The resulting map includes, in total, 299,171.31 km of roads in an area of nearly 4,000,000 km^2^, which is nearly double the length of other resources (the Digital Atlas of Roman and Medieval Civilizations road dataset is 188,555 km). This increase is due to a higher coverage of roads (Figs. 1b, 2a; e.g., the Iberian Peninsula, Greece, North Africa), but also by the decision to make a spatially explicit dataset that adapts routes to the geographical reality (i.e., to cross a mountain, our roads follow a winding pass rather than a direct line), resulting in a higher number of vertices for the road lines and higher total length (Figs. 1c; 2b). The dataset includes 14,769 road segments and consists of 14 connected components, reflecting the contiguous landmasses (including continental Europe, Britain, Asia–North Africa, and various Mediterranean islands). The core of the Roman terrestrial transport network were those roads classified as main roads (see Main/Secondary Roads) documented via milestones or historical sources. Our dataset reveals these covered 103,477.9 km (34.58%), and this number is unlikely to increase significantly, since these are the most documented and studied Roman roads. The dataset enables studies of how these main roads structured and enabled phenomena including Roman conquest and administration. Our dataset further includes 195,693.3 km (65.42%) of secondary roads which can be studied to understand the structure of more local mobility, and future data collection focusing on understudied areas could significantly increase this number. The dataset reveals for the first time that only 2.737% of the spatial location of the total road length is certain, whilst 89.818% is conjectured and 7.445% hypothesised (see Certainty categories). This shows a discrepancy between our knowledge of the existence and location of Roman roads: we know all of the included roads were used at some point during the Roman period, but their precise locations are not certain. Our confidence maps (see Technical validation) visualise this uncertainty and elaborate on it by expressing differences across the dataset in road density, spatial accuracy of road digitisation, and source reliability.

A major challenge is the absence of chronological evidence of the creation and change of roads, that is comparable at an Empire-wide scale. This means the current dataset cannot show the growth and change over time of Roman roads and the degree to which they built on and reused previously existing roads. Historical and archaeological sources teach us that transport networks grow organically, new roads are constructed on top of old ones, they change function and physical characteristics, and become disused. Detailed temporal evidence for road construction, use and change is only available for a handful of cases, making an evidence-based reconstruction of how the road system changed throughout the Roman period at an Empire-wide scale currently impossible. This should be the subject of dedicated large-scale efforts in future research.

Itiner-e makes such gaps in our current knowledge of Roman roads explicit for the first time, allowing this information to inform robustness tests and uncertainty quantifications of future studies, and specifying how and where future data collection efforts can improve our knowledge.

## Methods

**Fig. 3:**
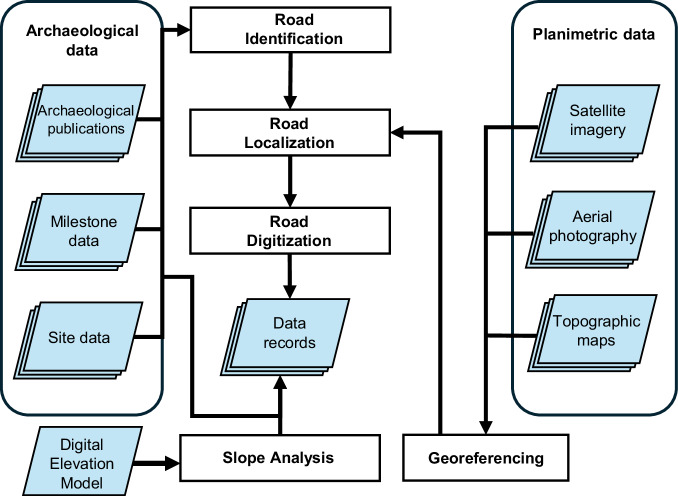
Workflow summarizing the data collection and digitisation process.

Road digitisation followed three steps (Fig. 3), with regional variation depending on the availability of sources and intensity of research activity, as well as collaborators (see Regional overviews): (1) identifying roads through relevant literature, historical and archaeological sources, gazetteers and milestones; (2) locating roads using modern and historical aerial photographs, modern and historical topographic maps, and modern and historical satellite imagery; (3) digitising roads using GIS, cross-checking with sources, and adding attribute information.

The first step consisted of identifying roads in sources and those revealed in prior studies within the research area, based on previously published atlases, regional summaries, surveys, milestones and excavations. Previous studies identified roads through a set of historical and archaeological parameters^19,20^, often based on a combination of historical and archaeological sources, diachronic studies of the landscape and the geographical characteristics of possible paths. A key factor in all prior research was the inclusion of roads on ancient itineraries such as the *Antonine Itinerary* or the *Tabula Peutingeriana*. The location of milestones, and the location of cities or settlements close to each other also allowed researchers to assume the existence of roads that connected them, since all ancient places must have been reachable by a terrestrial route. But only rarely have long stretches of Roman roads been excavated or survived to the present day. Key overviews of roads of the entire Roman Empire include the Barrington Atlas^8^, and its digital representation by the Ancient World Mapping Centre and by Mapping Past Societies, as well as the *Tabula Imperii Romani* (https://tir-for.iec.cat/), and summaries of the Roman road network in specific regions, provinces, countries or extensively surveyed areas (discussed per region in Regional overviews). This phase further included collecting and summarizing relevant archaeological and historical sources and publications for each region. Milestones were used as an important source to determine points where roads passed through, and for chronology, as they were placed along the roads to indicate the distance to ancient places and sometimes named infrastructure investments by emperors. Milestone data were collected primarily using the geocoded database LIRE (Latin Inscriptions of the Roman Empire)^21^, which includes 8,388 milestones with Latin inscriptions. While milestones with Greek inscriptions are also included in LIRE, they are not fully represented. The drawbacks of the sources used by LIRE include the absence of anepigraphic milestones, the imprecise or incorrect location of some milestones and missing or unknown chronological data. This necessitated case-by-case corrections by consulting additional region-specific sources of the milestone data (see per region in Regional overviews). Furthermore, milestones were not evenly distributed throughout the Roman Empire and their explanatory potential is limited mainly to the major public roads (*viae publicae*)^22^. Additional data about ancient places, sites and settlements was consulted from Pleiades, which is the gazetteer of ancient places, and Vici.org, the archaeological atlas of antiquity (https://vici.org/). The archaeological, milestone, and site data were used to identify the existence of individual roads and the general layout of the road network in each region.

In step two, we proceeded to spatially locate identified roads. The specific information obtained for each road in the previous step (location of settlements, location of extant remains from survey or excavations, location of bridges, milestones, road stations, etc.) was subsequently compared to different planimetric sources such as modern and historical aerial photographs (USAF 1950s photogrammetric flights^23^), modern satellite imagery (ESRI World Imagery^24^, Google Satellite^25^) and historical satellite imagery (Corona mission^26^), and modern and historical (19th–early 20th century) topographic maps (see a detailed overview of the sources for each region in Regional overviews). Available topographic maps were scanned and georeferenced as needed. This enabled locating the roads in the real world.

**Fig. 4:**
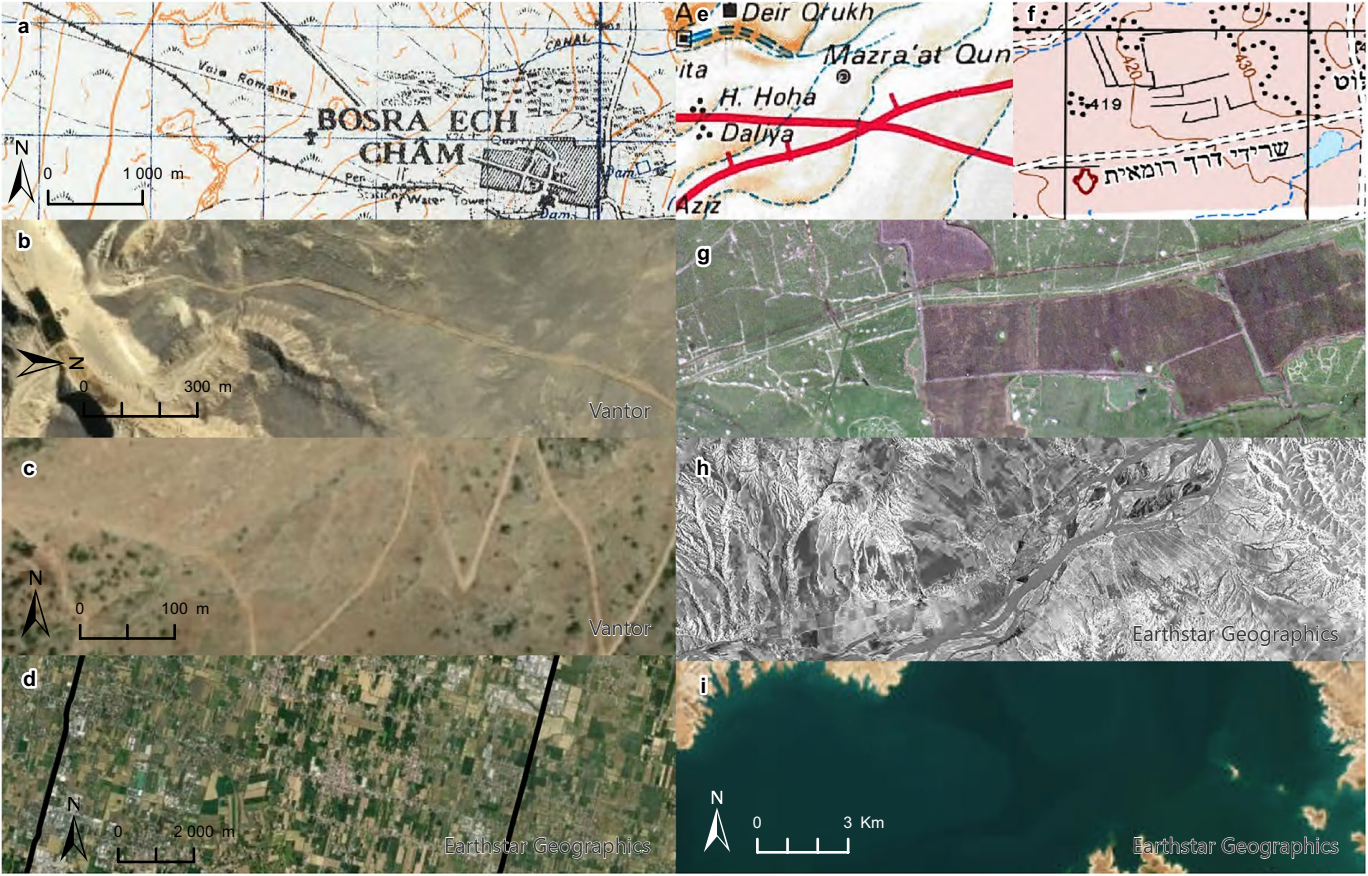
Examples of the road locating process. (**a**) A Roman road indicated as ‘Voie Romaine’ on a topographic map (Levant 1:50,000) leaving ancient Bostra (Bosra esh-Sham, Syria) due west; (**b**) Via Hadriana in the Eastern Desert of Egypt appears as a wide light linear feature on a darker soil on satellite imagery; (**c**) the Klimax pass between Argos and Mantinea (Greece) can be identified as a sharp switchback track on the mountain, partially overlaid by a modern wider track; (**d**) orthogonal Roman land divisions are retained by modern roads north of Padua, Italy; (**e**) a road drawn on the *Tabula Imperii Romani Iudaea/Palaestina* sheet; (**f**) the same area on the modern 1:50,000 topographic map of Israel, indicating ‘remains of the Roman road’ (in Hebrew), and (**g**) the same area on a modern orthophoto, where the Roman road appears as a straight line with sharp turns running roughly southwest to northeast across the picture. (**h**) An example of employing Corona satellite imagery for locating roads around the ancient city of Samosata (Samsat, Turkey) before flooding (Corona mission image DS1107-2138DA061-62), and (**i**) its current state.

In step three, each road section was manually digitised with a target spatial resolution of 5–200 m on a Geographic Information Systems (GIS) platform and stored as a single data record in a vector layer with an associated attribute table. A number of general principles or observations were followed to guide the digitisation work. In ideal cases, ancient roads can be visually identified on satellite imagery, aerial imagery, or are marked as such on topographic maps (Fig. 4a–g). These usually appear as straight linear features in flat areas (Fig. 4b), or as sharp switchback roads on slopes (Fig. 4c). Roman land divisions (centuriation), which include the construction of roads in an orthogonal layout, are often preserved in modern networks of roads and agricultural fields, especially in northern Italy^27^ (Fig. 4d), but also elsewhere (e.g., Tunisia^28^ and Syria^29^). All such identified features were cross-checked against the primary publications and additional datasets (milestones and site data) where applicable. However, in other cases where roads were not visible in imagery, the digitised road sections follow a line connecting known archaeological road remains, milestones, and ancient sites. The digitised paths of such segments are refined by comparing them to existing contemporary and historical roads on georeferenced topographic maps and satellite imagery. It is then possible to approximate the layout of a Roman road to one of the existing roads to achieve a more realistic road layout that takes into account local topography, especially in areas where more formidable obstacles are present (wetlands, rugged terrain, mountain passes, etc.). In many of these cases then, the conjectured line of a Roman road follows the line of a modern road, which is often corroborated by additional archaeological and historical evidence. The only modern roads which were excluded from consideration for corroboration with Roman roads were modern highways/expressways, which in most cases do not conform to any previous historical roads. This process results in a plausible digitised road course recorded as ‘conjectured’ or ‘hypothetical’ (see Certainty categories), which is informed by Roman remains and prior research whilst conforming to the geographical context. A consistent issue across all study areas was the presence of modern water reservoirs and dams. In these cases, we relied on historical topographic maps or historical satellite imagery (especially Corona satellite photography for the Near East, from ca. 1967–1972) taken before construction of any dams to digitise the road segments (Fig. 4h–i).

Several parameters were calculated for each road segment and added to the attribute table. Geodesic length in metres of each segment was calculated in GIS using the WGS 1984 World Mercator projected coordinate system (EPSG:3395). Average slope in degrees along the length of each segment was calculated over SRTM^30^ Global Digital Elevation Model with a resolution of 3-arc seconds. The dataset was finally cleaned to ensure consistency in terminology and topological continuity between line segments.

The work was performed as a collaborative effort between the co-authors of this paper undertaken between 2020 and 2024. The MINERVA (https://pure.au.dk/portal/en/projects/minerva-understanding-the-centuries-long-functioning-of-the-roman) project led the digitisation of the Roman road system in North Africa, the Near East, Asia Minor, and Southeastern Europe. The Viator-e (https://viatore.icac.cat/) project led the digitisation of the roads in the Western Roman Empire, excluding North Africa. The Viator-e project is a continuation of an earlier Mercator-e project, that focused on the Iberian peninsula (https://fabricadesites.fcsh.unl.pt/mercator-e/). These two projects’ efforts were supplemented with road datasets for the Eastern Desert of Egypt (ERC Desert Networks, https://desertnetworks.huma-num.fr/), Bithynia (Weissova), Rough Cilicia (Şahin), Pamphylia (Massa), Campania (Renda), the Dutch part of the Roman frontier (Verhagen), the Meuse and Scheldt basins (Bongers), and Britannia and Sardinia (Lewis). The data collection per region is described in Regional overviews.

### Research area

The geographical limits of the data collection are broadly defined by the extent of the Roman Empire during the Antonine dynasty, ca. 150 CE, when the Empire reached its largest extent (Fig. 1a). The inclusion of some regions does not strictly follow this chronological framework and is rather founded on available source material and natural connections of these regions to the Roman provinces in the 2nd century CE. Therefore, the road system on the left bank of the Euphrates between Samosata and Edessa (modern Samsat and Şanlıurfa, Turkey) is included, since the exact boundary between the Roman Empire and the Edessan kingdom is impossible to define exactly. Dura–Europos in Syria is included, although it was permanently annexed around the year 165 CE. Further regions to the east (Armenia, Mesopotamia) are not included as they were annexed by the Romans only for a short time in the early 2nd c. CE. A string of north Saharan oases (Jaghbub, al-Wahat, Marada, Zillah, Waddan and Fezzan in Libya, and Touggourt in Algeria) are included – even though they were not part of the Roman Empire they were part of the Roman trade network in North Africa. The region between Hadrian’s Wall and the Antonine Wall in Britain is also included, even though Roman control there was only ephemeral (ca. 142–182 CE).

### Chronological boundaries

CE (Common Era) and BCE (Before Common Era) are used throughout this paper and in the dataset. The lower chronological limit of the data collection is broadly defined by the first incorporation of each territory into the Roman dominion as a province (be it under the Republic or the Empire). Thus, the earliest dated road in the dataset is the Via Appia between Rome and Capua, built in 312 BCE. The lower boundary then varies on a regional basis (Table 1).

**Table 1:** Chronological period marking regions’ incorporation into the Roman dominion.

Incorporation of the areas under study into the Roman dominion	Region
300–200 BCE	Italy, Sicily, Sardinia, Corsica
200–100 BCE	Hispania, Greece, Africa (Tunisia, eastern Algeria, western Libya), western Asia Minor, southern Gallia, Adriatic coast
100–50 BCE	Crete, eastern Libya, western Algeria southern and northern Asia Minor, Cyprus, Syria
50 BCE–1 CE	Gallia (France), Germania (Benelux, Rhine valley), upper Danube, western Balkans, central Asia Minor, Egypt
1–50 CE	Central and eastern Balkans, Judaea, Mauretania (northern Morocco), Britannia
50–100 CE	Upper Euphrates, southern Syria
100–150 CE	Arabia (Jordan), Dacia (Romania)

However, the Romans usually incorporated older roads into their road system, which they then improved, paved, or equipped with bridges and milestones^20^. Therefore, evidence for the pre-Roman road network in each region was taken into account during data collection, based on available published data. Only in rare specific cases is it possible to establish a precise date for the construction of a completely new road. For this reason, the start date of most roads in the dataset is not fixed, and in such cases the date fields are filled with the value 9999, to indicate missing data (see the next section, Data field description). The upper chronological boundary for the data collection was set at 400 CE, as there were minor changes in the Roman road system until the early part of 4th century CE. That means we also only used milestones dated up to ca. 400 CE.

### Data field description

Each data record represents a road section between two ancient places or road crossings, and additional information associated with it (Table 2). Due to limited sources about many roads and limits to the scope of data collection, it was not always possible to assign all values to every data record. As a minimum, each data record contains ID, Name, Type, Citation, Bibliography, and Segment Certainty, in addition to Shape Length and Average Slope.

**Table 2:** Fields in the Itiner-e dataset.

Attribute	Value	Description	Example
FID	Numeric	Unique numerical identifier	3612
Name	Text	Start and end point of the road segment, ancient or modern toponyms	Caesarea Maritima–Legio
Type	Text	‘Main Road’ or ‘Secondary Road’	Main Road
Lower_Date	Numeric	Start date of the road, negative values for dates BCE. 9999 means uncertain.	70
Low_Date_E [Lower Date Error]	Numeric	Possible time span before the ‘Lower Date’ when the road might have existed. 9999 means uncertain.	1
Upper_Date	Numeric	End date of the road, negative values for dates BCE. 9999 means uncertain.	9999
Up_Date_E [Upper Date Error]	Numeric	Possible time span after the ‘Upper Date’ when the road might have still been in use. 9999 means uncertain.	9999
Descriptio [Description]	Text	Brief description	Conjectured part of the road through Carmel range
Citation	Text	Author performing the digitisation	Adam Pažout
Bibliograp [Bibliography]	Text	Short bibliographical reference (name, year) of the source(s) used for the digitisation	Tepper 2007, Paz and Paz 2006
Cons_per_e [Construction Period]	Text	Name of the magistrate or emperor during whose tenure the road was formalised, with exact dates in parenthesis	Vespasian (69–70 CE)
Itinerary	Text	Conventional name of the road in ancient or modern sources, ancient itinerary it was part of	Tabula Peutingeriana
Segment_s [Segment Certainty]	Text	‘Certain’, ‘Conjectured’, Hypothetical’	Conjectured
Shape_Leng [Shape Length]	Numeric	Geodesic length (in metres)	18158.767
Avg_Slope [Average Slope]	Numeric	Average slope along the length of the segment (in degrees)	4.03

Each record is identified by a unique numerical identifier and a descriptive name. The name is constructed from the two origin–destination place names of the digitised section, connected by a hyphen ‘-’. The ancient place name is used when known. The present-day toponym was used instead in cases where the name of the ancient settlement was unknown or did not exist. A characteristic geographical element was chosen as a place name in cases where the road ended without the existence of a settlement.

Further attributes are included for each road section related to the chronology of its creation and abandonment and, if known, the name of an emperor or a magistrate under whose mandate the road was built. Few roads can be dated with certainty, and it was outside of the scope of our data collection to obtain chronological information for all roads. These fields will be especially relevant to capture future new findings about road chronology, when the attributes will enable us to visualise the evolution of the Roman road system, or to compare the infrastructure investment of each Roman emperor.

Additional fields describe the type of road in the road system hierarchy (‘Main Road’ or ‘Secondary Road’), the creator of each data record, bibliography, field identifying the ancient name of the road or itinerary it is part of, and segment certainty (‘Certain’, ‘Conjectured’, and ‘Hypothetical’). Additional fields describe physical properties of the road segments – length (in metres), and average slope (in degrees). Each entry also has a URI on the live itiner-e.org version of the dataset, which enables linking to other online resources.

### Definition of road section/record

For the purposes of the data collection, a road was defined as any line of terrestrial communication connecting sites existing within the defined geographical and chronological boundaries. This definition includes both formal (built, engineered) and informal (non-built) roads, i.e., both paved and unpaved roads which are in regular use as an accepted line of communication (e.g., desert camel tracks). Every inhabited place in the Roman world must have been reachable within its continental or island terrestrial context by a path of some sort, but road digitisation was not attempted in cases where no information was available for tracks and pathways leading to an inhabited place.

In the dataset, all roads are represented as line (vector) segments, where each segment corresponds to a single data record. A road segment spans from an intersection with another segment to the next intersection with a different segment, or from a starting point to the nearest intersection. This means that a single named road (e.g., Rome–Capua) will be split into multiple segments as the road is intersected by other roads along its course. These multiple segments will share many attributes (name, type, author, etc.) but not all of them – Shape Length and Average Slope are almost always unique, and the FID is a unique identifier for each record.

### Main/Secondary Roads

A basic road hierarchy is provided by designating each road segment as either a ‘Main Road’ or ‘Secondary Road’. The assignment of these categories to the road segments is for the most part based on prior designations by archaeological and historical publications used in the digitisation process (see further below). We used the following principle in cases where prior designations were lacking. A road segment is defined as a ‘Main Road’ if it has more than one of these characteristics: (a) the presence of milestones, (b) is part of an ancient Itinerary (chiefly the *Antonine Itinerary* and *Tabula Peutingeriana*), (c) it shares (a large part of) its course with a historically known major road indicated on 19th/early 20th century maps. The remaining roads were classified as a ‘Secondary Road’. This two-tier hierarchy is satisfactory for most of the study area, although in several regions with detailed road data an additional third tier could have been used (e.g., the region of Sicyon in Greece, Sagalassos in Turkey, Durocortorum–Reims in France, etc.), but due to the limited extent of these regions this was not implemented.

### Certainty categories

The segment certainty specifies the spatial accuracy and confidence in the digitised location of the road segment. Three values are defined: ‘Certain’, ‘Conjectured’, and ‘Hypothetical’. ‘Certain’ designates well-documented segments in our sources that were digitised with high spatial accuracy (less than 50 m deviation in the mountainous terrain, less than 200 m in the plains). Most roads fall into the ‘Conjectured’ category, i.e., identified road segments with lower spatial accuracy due to lower level of documentation in our sources. ‘Hypothetical’ is reserved for identified but not located roads, or identified roads where the physical infrastructure of the roads was less fixed or where multiple parallel tracks might have existed (e.g., desert areas, flood plains). In addition, ‘Hypothetical’ is used for roads which are speculated to have existed in antiquity but insufficient evidence exists to classify them as either ‘Certain’ or ‘Conjectured’.

### Regional overviews

The following section provides an overview summarised by region of the main sources used in the processes of identifying and locating roads and in specific cases methods used in the process of digitising roads.

#### North Africa

For Morocco, the main source was the *Barrington Atlas*^31^, supplemented by a few other studies^32,33^. Except for a road intersection at the site of ancient Volubilis (Fertassa) which is visible on the satellite imagery, all roads are only conjectured, following existing roads. The primary topographic map used for digitisation was a 1:200,000 map from the Army Map Service^34^. In comparison to previous datasets, this work resulted in improving the spatial accuracy of the road data.

Algeria, Libya, and western Libya (Tripolitania) is covered by a map by Pierre Salama^6^. This source was supplemented by a study on the region of Sitifis (Sétif)^35^. Data on ancient sites from Pleiades and Vici were corroborated with the *Atlas Archéologique d’Algérie*^36^. Milestone data in LIRE were cross-checked against the *Corpus Inscriptionum Latinarum*^37^, but their interpretation and the assignment of construction dates to roads was found to be beyond the scope of the present research and only a few roads were unambiguously dated. The data for Tunisia is more detailed, with up-to-date research on several roads (Carthage–Theveste, Capsa–Tacape, etc.)^38^. Further roads could be digitised using an atlas of Roman land divisions (centuriation), which marks several Roman roads on modern 1:50,000 topographic maps^28^. A number of these roads could have been digitised with high accuracy as it was possible to visually identify them on modern satellite imagery. The majority of roads tend to follow a variety of modern roads, field roads, and tracks marked on topographic maps and corroborated on modern satellite imagery. Primary topographic maps used for Algeria were 1:50,000, and 1:200,000 Army Map Service maps, and for Tunisia, 1:50,000, 1:100,000, and 1:200,000 Army Map Service maps^39–43^. In comparison to previous datasets, this work resulted in improving spatial accuracy across the region, and increased coverage in northern Tunisia, the desert region, and Algeria.

The primary source for Libya was Mattingly^44^ and two sheets of the *Tabula Imperii Romani*^45,46^, and the outlines of the main Roman roads and their milestones are provided in *Inscriptions of Roman Tripolitania*^47^ and *Inscriptions of Roman Cyrenaica*^48^. A study of roads in the hinterland of Lepcis Magna (el-Khoms)^49^ and Cyrene^50^ provided detailed data that could be corroborated with modern satellite imagery and topographic maps, resulting in high accuracy of digitisation for the two cities. The road network for the Fezzan region was reconstructed based on the *Archaeology of Fazzan*^51^. Roads around Bani Walid, Misrata District, mostly follow wadi beds, along which settlements and water sources are concentrated, occasionally crossing rocky plateaus where indicated on modern topographic maps. However, their exact course, as with other desert roads, is hypothetical. Coastal roads in both Libya and Tunisia avoided *sabkhas* – seasonally inundated coastal salt pans not suitable for travel. Principal topographic maps used for coastal Tripolitania and Cyrenaica were 1:100,000 maps produced by the Army Map Service^52,53^. The desert area was covered by 1:250,000 maps by the Army Map Service^54^. In comparison to previous datasets, this work resulted in increased spatial accuracy and increased coverage in Tripolitania and the city of Cyrene.

In Egypt, many desert routes were identified on both modern topographic maps and satellite imagery, following the approach to the identification of caravan routes outlined by Bubenzer and Bolten^55^. A specific problem is presented by the Nile Valley and its delta, which traditionally relied primarily on water transport. Both the *Barrington Atlas*^56,57^ and the *Tabula Imperii Romani*^58^ suggest a land route along the Nile and the delta, based on ancient sources (the *Tabula Peutingeriana* and the *Antonine Itinerary*); therefore, these roads were included. However, due to the regular flooding of the Nile and numerous waterworks in the valley (irrigation channels, dykes), the location of these roads is very uncertain and they were likely located closer to the edges of the Nile Valley (such as modern roads). The main reference work used for the Western Desert and Sinai was Paprocki^59^. Primary topographic maps used for Egypt were 1:250,000 maps by the Army Map Service^54^. In comparison to previous datasets, this work resulted in increased spatial accuracy and increased coverage in the Eastern Desert, Western Desert and the Nile Valley.

The road system in the Eastern Desert included in our dataset was developed by the ERC Desert Networks project (https://desertnetworks.huma-num.fr/). Our knowledge about these routes came from the *Tabula Imperii Romani* (sheet 36 Coptos)^58^, supplemented by regional surveys^60,61^ and archaeological work on the stations of the Coptos–Myos Hormos road^62^ and the Coptos–Berenike road^63^. However, the roads themselves and their exact routes were not well known as there are, e.g., no milestones. To overcome this lack of data, the Desert Networks project has adopted an original, semi-empirical approach, combining 1:50,000 scale maps from the Egyptian Survey Authorities, a DEM with a spatial resolution of 11 m, a corpus of 288 archaeological sites with precise locations, and 60 reconstructed itineraries by modern travellers (18th–19th centuries) who travelled through the desert in conditions comparable to Roman ones (environment, transport means and logistics)^64^. These data were used to calibrate various factors involved in camel caravan travel and to validate the least-cost routes calculated between sites. The modelled network was then used to generate routes, taking into account transport infrastructure, navigation conditions, difficulties of the terrain and the topographical constraints specific to camels. The Roman roads were reconstructed by calculating the least-cost paths^65,66^ between the known Roman road stations according to ancient sources (the *Tabula Peutingeriana* and the *Antonine Itinerary*, and ostraca found at the stations^67^). The occupation of most of them is well-dated thanks to recent excavations. It was thus possible to refine the route and propose an itinerary for each 50-year period between 1 CE and 300 CE, allowing us to consider the evolution of the network over time. The ERC Desert Networks project data were adapted to our data structure (removal of multiple overlapping segments, merging of segments diverging less than 200 m from each other, adjustment of start and end point vertices to archaeological sites).

#### The Near East

The principal source for the southern Levant (Israel, Jordan, Palestinian Territories, southern Syria) is the *Tabula Imperii Romani*^68^, supplemented by numerous detailed studies on individual roads (Legio-Scythopolis^69^, Jerusalem-Jaffa^70^, Via Nova^71–73^, Gaza-Petra^74^), cities (Caesarea^75^, Jerusalem^76^, Petra^77^), and regions (Galilee^78^, southern Syria^79^, Negev^80^, north-east Jordan^81^). Many of the Roman roads were in continual use until the 20th century and are clearly marked on 19th/early 20th century maps, such as the *Survey of Western Palestine*^82^, or the 1:50,000 Levant map series by the French Army^83^. Many are still used to this day as modern paved roads or local field roads and tracks. In the case of Israel and the Palestinian Territories, high-resolution orthophotos and detailed 1:50,000 modern topographic maps were available on the Israeli government portal GovMap (https://www.govmap.gov.il/), which enabled digitisation with high spatial accuracy and resolution. Jordan was mostly covered by the French Army 1:50,000 Levant series and 1:250,000 topographic maps by the Army Map Service^83,84^. In comparison to previous datasets, this work resulted in increased spatial accuracy and better coverage in the desert and mountainous regions.

A key overview of Lebanon and Syria is the study by Thomsen^85^ and by Bauzou^86^. The Syrian desert was covered by research by Antoine Poidebard and René Mouterde^87,88^. Northern Syria and southeastern Turkey was covered in the *Tabula Imperii Byzantini project*^89^ and Comfort^90^. Limited detailed studies were dedicated to only a few areas, mainly the city of Apamea^91^ and Antioch^92^. As in the southern Levant, many Roman roads have been used continually, corresponding to modern paved and unpaved roads marked on topographic maps (1:50,000 French Army series^83^) and visible on modern satellite imagery. The road system in flooded areas of the Atatürk and Assad dams was digitised over Corona satellite imagery and historical topographic maps recording the situation before the construction of the dams. The maps produced by Poidebard and Mouterde were corroborated with historical topographic maps (1:200,000 by the French Army^93^) and modern satellite imagery. The area between the Euphrates and the Taurus Mountains was covered by 1:100,000 military maps by the USSR General Staff^94^. In comparison to previous datasets, this work resulted in increased spatial accuracy and better coverage in the Euphrates valley, and desert and pre-desert areas.

The roads of ancient Cyprus were described in detail by Bekker-Nielsen^95^, and a detailed survey was conducted on the Akamas Peninsula^96^. Most roads could be corroborated with historical roads recorded in Kitchener’s survey of Cyprus (1:63,360)^97^ and modern topographic maps (1:50,000) by the Army Map Service^98,99^. Several roads not recorded on these maps (mainly on the Akamas Peninsula) could be identified on modern satellite imagery. In comparison to previous datasets, this work resulted in increased spatial accuracy and increased coverage across the island.

#### Asia Minor

The principal source for the main Roman roads and their milestones in Asia Minor was the work by David French^100–107^ and the *Tabula Imperii Byzantini*^10,12–16,108^. This general survey is supplemented by various regional studies for the Euphrates frontier^109^, Cappadocia^110,111^, Pontus^112^, Lycia^113^, Caria^114^, and the Küçükmenderes valley^115^; and by further studies focusing on individual roads (Pilgrim’s road^116,117^) and cities (Tavium^118,119^, Neoclaudiopolis^120^, Pergamum^121^, Aphrodisias^122^, and Sagalassos^123^).

Many Roman roads were in continual use until the 20th century and thus could be corroborated with Kiepert’s *Karte von Kleinasien* (1:400,000)^124^ and the *Deutsches Heereskarte* (1:200,000)^125^ and with modern satellite imagery. The remainder of the roads mostly follow modern paved and unpaved roads as recorded on 1:100,000 USSR General Staff topographic maps^94^. A group of abandoned historical roads likely corresponding to Roman roads were identified on modern satellite imagery principally along the Euphrates frontier (the Melitene–Satala road) and in Lycia (the Patara–Phellos road above Kalkan, etc.). They were digitised in places where these could be corroborated with additional archaeological and historical data. In places where both historical and modern topographic maps lacked roads that could guide digitisation, a preference was given either to valley bottoms or ridges as suitable places for unobstructed travel. Particular problems occurred in digitising roads in dynamic fluvial environments where coastal progression and silting occurs. It necessitated using georeferenced maps published by geoarchaeological research in the Büyük Menderes^126,127^, Küçükmenderes^128^, and Dalyan^129^ river valleys. Detailed archaeological reports and visible remains identifiable on modern satellite imagery permitted the road network to be digitised in the vicinity of several cities with very high accuracy and detail (Ephesus, Magnesia^130^, Pergamum, Milet, Laodicea, Sagalassos, Tavium).

Mountain roads in Pisidia and Pamphylia (Termessus, Selge and Etenna, Antalya Province) were supplied as GPS tracks by Michele Massa and adapted to our data structure. These roads were digitised as part of a project aimed at creating a new archaeological trail in the region, and for the most part have not been published before with the exception of the ‘Döşeme Boğazı’ (Via Sebasteia)^131^. In comparison to previous datasets, this work resulted in increased spatial accuracy and increased coverage, especially in western and north-eastern Asia Minor.

##### Bithynia

The road data for Bithynia were directly supplied by Barbora Weissova^132^ and adapted to our data structure. The first descriptions of Roman roads in Bithynia were published in the 19th century^133–136^. A fundamental contribution to the study of the road system represents W. M. Ramsay’s *The Historical Geography of Asia Minor*^137^.

Bithynia was explored in the last two decades of the 19th century by von Diest, von der Goltz and Anton^138–142^. Results of their travels are particularly helpful for the present study as they brought to light 28 remains of pavements of roads and nine bridges with meticulous descriptions of their geographic positions, allowing for the rectification of the courses of ancient roads.

Researchers in the 20th century focused mainly on the reconstructions of the courses of the supra-regional Pilgrim’s Road^116,143,144^ and the regional ‘Northern Road’^145,146^, leaving the course of the Pilgrim’s Road in Nicomedia and following the eastern direction through Prusias ad Hypium-Claudiopolis–Cretia Flaviopolis–Hadrianopolis. The development of the roads from the Early Byzantine period onwards was examined by Belke^108,147,148^ and Avramea^149^.

Reconstructions in the present study were furthermore based on nodal points and roads compiled in the *Barrington Atlas*^150^, French’s^103^ compendium of milestones in Pontus and Bithynia, and information about remains of roads and bridges scattered among regional archaeological and epigraphic studies and reports^151–159^. In comparison to previous datasets, this work resulted in increased spatial accuracy and increased coverage in the Marmara region.

##### Rough Cilicia

The milestones from Cilicia were first compiled by Theodor Mommsen in 1873 as part of CIL III^160^, in 1902 expanded together with Hirschfeld and Domaszewski, with milestones from Pamphylia and Cilicia. Additional milestone inscriptions in Cilicia were collected by Keil and Wilhelm^161^, and by Mitford and Bean^162,163^. French published several studies on local finds^164–168^ and a new catalogue of milestones^169^. In 2014, French provided a revised monograph, which includes new milestones from Cilicia^106^.

Sayar’s essay published in 2002 on the ancient road connections of Cilicia in the Roman imperial period provided a repetition that was enriched with some milestone inscriptions, listed in *Tabula Imperii Byzantini* 5, of the road connections of Cilicia^170^.

Information from this prior research was combined with new surveys in Rough Cilicia (the regions of Anemurium-Anamur and Diocaesarea/Olba-Uzuncaburç), which began in 2011, to collect and publish milestone inscriptions from the area. During the fieldwork, several new Roman roads, showing connections between ancient cities, as well as new milestones, were discovered^171–174^, and previously published inscriptions were re-analysed and re-interpreted in some cases. The identified roads were recorded with GPS and entered into a topographic map (MapSource TOPO). To compile the milestone inscriptions, a database was developed using FileMaker Pro database software, in which the inscriptions were already entered. GPS tracks were adapted to our data structure and incorporated into the dataset. In comparison with previous datasets, this work resulted in increased spatial accuracy and increased coverage in the region.

#### Southeastern Europe

Most of Greece, European Turkey, and parts of Bulgaria were covered by the *Tabula Imperii Byzantini*^9,11,175,176^. The *Tabula Imperii Romani* covered parts of Greece, Albania, North Macedonia, Kosovo, and southern Serbia^177–179^. Several maps from the *Barrington Atlas* were used as a basis to digitise especially secondary roads in Dacia (Romania), Moesia Superior and Moesia Inferior (Serbia and Bulgaria)^180,181^.

Detailed studies for Greece covered mainly the western Peloponnese^182,183^, including individual city territories (Corinth^184^ and Sicyon^185^), Attica^186–188^, Thessaly^189^, southern Euboea^190^, and lower Macedonia^191^. Pritchet described several individual roads^192–194^. The Via Egnatia, spanning from the Adriatic to the Bosporus, attracted most scholarly attention^195–199^. It was possible to corroborate most of the roads with the known historical roads presented on the *Carte der Europaeischen Tuerkey* (1:575,000)^200^, *Carte de la Morée* (1:200,000)^201^, *Carte de la Grèce* (1:900,000)^202^, *Karten von Attika* (1:25,000)^203^, and modern topographic maps (1:50,000 USSR General Staff)^204^, since many of these were used until the 20th century or they survive as local roads and tracks. In several cases, it was possible to identify the ancient roads on modern satellite imagery. Pikoulas^182,183^ provided precise GPS coordinates of the remains of roads which were used for digitisation. In other cases, the roads follow modern paved and unpaved roads and tracks, marked on modern topographic maps and visible satellite imagery. Several roads in the central Peloponnese (Arcadia) connecting Pheneos, Orchomenos, and Mantinea were added as hypothetical (based on the *Carte de la Morée*), since they must have existed in the Roman period. Coastal progression re-shaped the landscape in the deltas of several rivers, and georeferenced maps stemming from geoarchaeological research were used in these cases: the Vardar river^205,206^, the Sperchios river^207,208^, the Acheron river^209^ and the Kalamas river^210^. The road network of Crete should be treated as preliminary, since only limited studies were done on the cities of Aptera and Hierapytna^211,212^. The rest of the road network was reconstructed by connecting identified sites from the *Tabula Peutingeriana* with known and still existing historical roads identified by Pendelbury^213^ and corroborated on 1:50,000 British Army topographic maps. In comparison to previous datasets, this work resulted in increased spatial accuracy and increased coverage in Attica, the Peloponnese, Acarnania, and Epirus.

The Roman and ancient road network in Albania was investigated mainly in its southern part^214^, along the Via Egnatia^198^, and in the city of Butrint^215,216^. For the remainder of the roads, the main reference was the *Barrington Atlas*^180^. Most of the digitised roads follow modern paved and unpaved roads and tracks found on the 1:50,000 topographic maps (USSR General Staff)^217^ and corroborated with modern satellite imagery. In comparison to previous datasets, this work resulted mainly in increased spatial accuracy and increased coverage in the region of Butrint.

A principal overview for Roman roads in Serbia and Kosovo was Petrović^7^, more detailed albeit limited surveys were conducted around Viminacium (Kostolac, Serbia)^218^, Ulpiana (Gračanica/Graçanicë, Kosovo)^219^, and on the road Naissus (Niš, Serbia)–Ratiaria (Arčar, Bulgaria)^220,221^. For a variety of secondary roads, the main source was the *Barrington Atlas*^180^ and the *Tabula Imperii Romani*^177^, which were also principal sources for Northern Macedonia. All of the roads could be corroborated with historical and modern roads found on both historical and modern topographic maps (*Yugoslavia* 1:100,000 by the War Office, 1:575,000 *Carte der Europaeischen Tuerkey*, and 1:864,000 *General-Karte der Europaeischen Turkei und des Konigreiches Griechenland*)^200,222,223^ and identified on modern satellite imagery. In comparison to previous datasets, this work resulted in increased spatial accuracy and increased coverage in several areas along the Danube (e.g., Viminacium).

An overview of Roman roads in Bulgaria was provided by Madžarov^224^, which was supplemented by a number of studies focusing on Via Militaris^225^, and the region of the middle Strymon river^226^. The lower Danube was treated by Panaite^227^ and Ţentea *et al*.^228^, and the geoarchaeological reconstruction of the landscape surrounding ancient Histria (Istria, Romania) was used to reconstruct the roads approaching the city^229^. The only additional source for European Turkey, besides the *Tabula Imperii Byzantini*, was a study of a ‘North Road’ by Karaca^230^. Many of the Roman roads remained in continual use until the 20th century and it was possible to corroborate them with historical topographic maps (*Bulgaria* 1:126,000, 1:575,000 *Carte der Europaeischen Tuerkey*, and 1:864,000 *General-Karte der Europaeischen Turkei und des Konigreiches Griechenland*)^200,222,231^ and modern topographic maps (1:100,000 USSR General Staff)^232^. Some roads that do not appear on these maps (mainly along the lower Danube and the Danube delta) were digitised using modern paved and unpaved roads and tracks that connect the ancient sites obtained from modern satellite imagery. In comparison to previous datasets, this work resulted in increased spatial accuracy and increased coverage in all areas south of the Danube.

The Roman roads of Dacia (Romania) were treated by Fodorean^233,234^. The data on Roman sites was improved by consulting the dataset of archaeological sites from the *National Archaeological Repertory* (RAN) of Romania (http://ran.cimec.ro/). Most of these roads were corroborated with historical topographic maps (1:200,000 by Austria-Hungary military mapping), modern topographic maps (1:100,000 *USSR General Staff*)^235,236^, and modern satellite imagery. The roads typically follow the course of modern paved and unpaved roads, and tracks. In comparison to previous datasets, this work resulted in increased spatial accuracy and increased coverage in the Transylvanian plateau.

#### Italy

The roads in central Italy have Rome as a focal point, they are numerous, many are named and are well known^237^. The roads published in the *Barrington Atlas* synthesised the previously published information and were used as a starting point. It was supplemented by the works of Crainz and Giuliani^238^ and Valenti^239^. The digital documentation offered by the *Appia Regina Viarum* (http://appia.beniculturali.it/appia/) project has been used to reconstruct the course of the Via Appia. Works used to identify possible road layouts in Northern Italy include, Bosio^240^ and Mateazzi^27^ for the Venice area, Page^241^ and Luccardini^242^ for the Po valley and Liguria, and Nouvel and Cramate^243^ for Alpine routes, in addition to the *Barrington Atlas*^244,245^. Finally, most of the Roman roads of Puglia, Basilicata and Calabria have been digitised following the *Barrington Atlas*^246,247^ and using sources such as Zocco^248^ and Ceraudo^249^. In comparison to previous datasets, this work resulted in increased spatial accuracy and increased coverage in the Alpine piedmont.

##### Northern Campania and the middle Volturno valley

The road data is based on the work by Prof. Giuseppina Renda^250–253^, which in turn is based on a diversity of sources: archaeological documentation, fieldwalking and direct knowledge of the territory, combined with literary and ancient itineraria, epigraphic, ancient and contemporary cartographic sources, and supported by remote sensing and spatial analysis. The dataset originates from field surveys of the tracks, combined with the digitisation of ancient cartography and the results of photo interpretation and archaeological excavation. In comparison to previous datasets, this work resulted in increased spatial accuracy and increased coverage in Campania.

##### Sardinia

The location of Roman roads in Sardinia was digitised using the Roman road system as presented by Attilio Mastino^254^ and digitised by Lewis^255^. Roman roads were predominantly identified from excavation and survey data. Where possible, *in-situ* milestones were also utilised. In comparison to previous datasets, this work resulted mainly in increased spatial accuracy.

#### Gallia

In Narbonensis (southern France), one of the most important roads was the Via Domitia, which has been studied by Laforgue *et al*.^256^. The territory and the roads from this region have been studied by Leveau^257^. The eastern part of this region was published by Leveau and Segard^258^ (after Benoit^259^), while Passelac^260^ has been working on the route of the Via Aquitania. In the region of Aquitania, we relied on data from the Aquitaviae research group, who conducted a field survey of Roman roads (https://aquitaviae.hypotheses.org/). Some of the most recent sources used to digitise Roman roads in this region have been Baret^261^ and Cribeller^262^. For completing the Lugdunensis province (northern and central France), the work of Bayard and Lemaire^263^, Cloppet^264^, Cribellier^265^, Ferdière, Monnier and Cassard were used^266^. Nouvel and Venault^267^ studied the Roman roads of the Alps. Provost^268^ and Taboué^269^ were primary sources for north-east of France. For the region of Bretagne, we also have used the information provided by the Voies Romaines de Bretagne (https://voies-romaines-bretagne.com/index.html). In comparison to previous datasets, this work resulted in increased spatial accuracy and increased coverage in northern France and Belgium.

#### The Iberian Peninsula

As general sources, we have used Arias’ publication about all Roman roads of the Iberian peninsula^270^, completing certain areas with information from the *Barrington Atlas*^271–274^. The northeastern territories have been studied in detail by de Soto^275^. Magallón^276^ published all the Roman roads of the central and western Pyrenees and Pre-Pyrenees regions. Arasa and Rosselló^277^ published a book about the Roman roads of the western coast of the peninsula. The Roman roads of the Baetican province have been studied by Corso^278^ and Sillières^279^. Lusitania and the Portuguese territories were studied by Mantas and Alvarez Martinéz^280^. For the territory of Castilla y León, the principal source was Moreno Gallo (https://www.viasromanas.net/). Finally, to complete the Northwestern part of the Iberian peninsula, we used the work done by Argüelles-Álvarez^281^, Güimill-Fariña and Parcero-Oubiña^282^, Losada Pérez^283^ and Rodríguez Colmenero^284^. In comparison to previous datasets, this work resulted in increased spatial accuracy and increased coverage across the whole of the Iberian peninsula.

#### Germaniae, Raetia and Noricum

The principal reference source for the digitisation of roads in the upper Germanian and upper Danubian provinces (Raetia, Noricum) and the Alps was the *Barrington Atlas*^244,285^ and *Corpus Inscriptionum Latinarum*^286^, which also provided data on the milestones and the itineraries. The communication and exchange networks in the Alps were examined by Flügel *et al*.^287^. The routes starting in the Po valley and leading up to the Alpine passes were examined by Bosio^240^. In comparison to previous datasets, this work resulted mainly in increased spatial accuracy.

#### Pannonia

The principal reference source for the digitisation of roads in Pannonia (roughly modern day Hungary, on the left bank of the Danube and eastern Austria) was the *Barrington Atlas*^288^. A comprehensive description of the Limes road and associated sites was provided by the Danube Limes project, conducted in the context of inscribing the Danubian Limes as a UNESCO World Heritage Site^289,290^. Recent research concentrated on the site of Brigetio (Komárom, Hungary)^291^. The local road network in the hinterland of Savaria (Szombathely, Hungary) was briefly examined by Borhy *et al*.^292^ and around nearby Arrabona (Győr, Hungary) by Teichner^293^. The road network around the legionary camp and the civil settlement at Carnuntum (Petronell and Bad Deutsch-Altenburg, Austria) was based on the published maps by Gugl *et al*.^294^. In comparison to previous datasets, this work resulted in increased spatial accuracy and increased coverage in the region of Carnuntum.

#### Lower Germania, the Dutch part of the Roman frontier

The reconstructions of the Dutch Roman road system south of the Rhine are based primarily on registered observations in the Dutch national archaeological database system, ARCHIS (https://archis.cultureelerfgoed.nl/), and are supplemented with information from the Dutch national repository for archaeological research data, *DANS DataverseNL* (https://dataverse.nl/dataverse/dans/). While usually quite accurate in terms of location, many observations have low degrees of accuracy when it comes to dating, simply being referred to as ‘Roman’. Establishing an end date for the observed segments is often especially problematic. Coverage is best for the western part of the Rhine frontier (from Utrecht to the North Sea) and in the province of Limburg (the valley of the Maas and the so-called Via Belgica). Conjectured and hypothesised road sections are mostly based on maps and descriptions in the secondary literature^295–298^. These mostly rely on archaeological, historical and topographical analysis, and their accuracy can often not be assessed. Notably, reconstructions for the southwestern part of the Netherlands (Zeeland, parts of Zuid-Holland and most of Noord-Brabant) are either absent or have no underlying evidence, due to the major changes of the landscape since the Roman period. No additional modelling or reconstruction was attempted. In comparison to previous datasets, this work resulted in increased spatial accuracy and increased coverage along the Rhine.

#### Lower and Upper Germania, Gallia Belgica, and the Meuse and Scheldt basins

For the Scheldt and Meuse basins, the project used the detailed reconstructions by M.-H. Corbiau^299–303^, supplemented by various regional studies^297,304–309^. Despite a large amount of data, several regions remain underrepresented, such as the (Campine) Meuse-Demer-Scheldt area. Notably, the dataset exclusively relies on digitisation of published sources and does not represent additional analyses. The data was compiled as part of the project Inland Waterways in the Roman Transport Network of the Gallic and Germanic Provinces (c. 50 BC–c. AD 400, https://research.flw.ugent.be/en/projects/inland-waterways-roman-transport-network-gallic-and-germanic-provinces). In comparison to previous datasets, this work resulted in increased spatial accuracy and increased coverage in north-eastern France and Belgium.

#### Britannia

The reconstruction of the Roman road system in Britain is primarily based on recorded observations by Margary^310^. Additional roads in the south-east midlands of Britain are conjectured by the Viatores group^311^. These two sources were digitised by Bishop^312^. To create an interconnected network, a number of road sections were connected using straight lines^313^. In comparison to previous datasets, this work resulted mainly in increased spatial accuracy.

### Data cleaning

A data cleaning phase was designed for this collaborative data collection effort, which included three steps: 1) ensuring consistent use of standardised terminology across data records; 2) proofreading and corrections of all textual fields; 3) checking for topological continuity and identifying topological errors in the vector polyline layer containing road segments. First, standardised terminology was applied to all fields following Table 2 (e.g., ‘Main Road’/’Secondary Road’, ‘CE’/’BCE’, etc.). Second, all ancient place names were corrected to conform to the Latin and Greek versions as recorded in the *Barrington Atlas of the Greek and Roman World*^8^ and the Pleiades dataset. Modern place names were derived from modern topographic maps, but omitting diacritic marks and other grammatical markings. Diacritics were retained only for personal names in the ‘Citation’ and ‘Bibliography’ fields. Third, the vector geometry of all road segments was checked, so that all are cut to intersections with other roads and are connected with (i.e., snapped to) vertices at the intersections. Continuity of road names and itineraries was checked, so they do not contain gaps or are not otherwise disconnected (e.g., the ‘Via Appia’ will be presented as a continuous line of vector segments, same as all roads belonging to the ‘Tabula Peutingeriana’ itinerary). The data cleaning was performed manually in GIS by a small team of three people over the course of four months.

## Data Records

The dataset is available in an open Zenodo repository^314^ with a canonical citation^315^. It contains 14,769 data records (road segments) and is stored as a polyline vector layer in the shapefile format (itinere_roads.shp), as a geopackage (itinere_roads.gpkg), and as a geojson database (itinere_roads.geojson). The shapefile and geopackage is projected onto the WGS 1984 World Mercator (EPSG:3395) coordinate system, while the geojson database is in the WGS 1984 (EPSG:4326) coordinate system. The Zenodo repository was chosen over a subject-specific repository to encourage findability and reuse beyond the field of archaeology, given the cross-disciplinary value of the dataset. The total size of the shapefile and associated files is 144 MB; the geopackage is 33.62 MB; the geojson database is 78.05 MB. The files can be opened in any GIS software or spatial database. Each data record contains only a shortened bibliographical reference (name and year), and all full references are stored in an openly accessible Zotero bibliography (https://www.zotero.org/groups/5141113/itiner-e) and attached to the Zenodo repository in bibtex format (.bib). The unchanging version of the dataset as described in this article is stored on Zenodo^314^ with a canonical citation (https://itiner-e.org/).

## Technical Validation

The available sources differ significantly between regions of the former Roman Empire due to preservation and research biases (see Methods). Some areas have good preservation of traces of roads in the landscape, while in others it is more obscured due to land use and building activity; some areas received vast amounts of research attention in the form of surveys and excavations, and others have not. The accessibility to the authors of sources and local knowledge from the roughly 40 countries in Europe, Africa and Asia that lie inside the boundaries of the former Roman Empire has also put practical limits on completeness. Some areas concern a significant increase in the number and accuracy of roads (e.g., the Iberian Peninsula, Egypt, Greece), others a significant increase in accuracy (e.g., the southern Levant), and yet others remained more similar to previous digitisations but with added metadata (e.g., some Central European regions). This has resulted in heterogeneities across the dataset in road density, spatial accuracy of road digitisation, and source reliability.

**Fig. 5:**
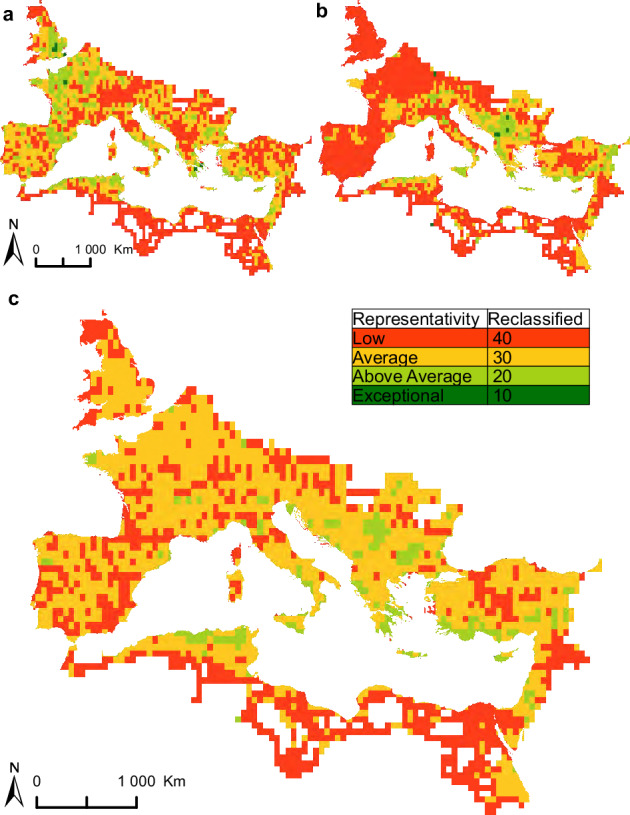
The representativity map (**c**) is created by numerically reclassifying and summarizing the (**a**) road density map and (**b**) vertex count map.

**Fig. 6:**
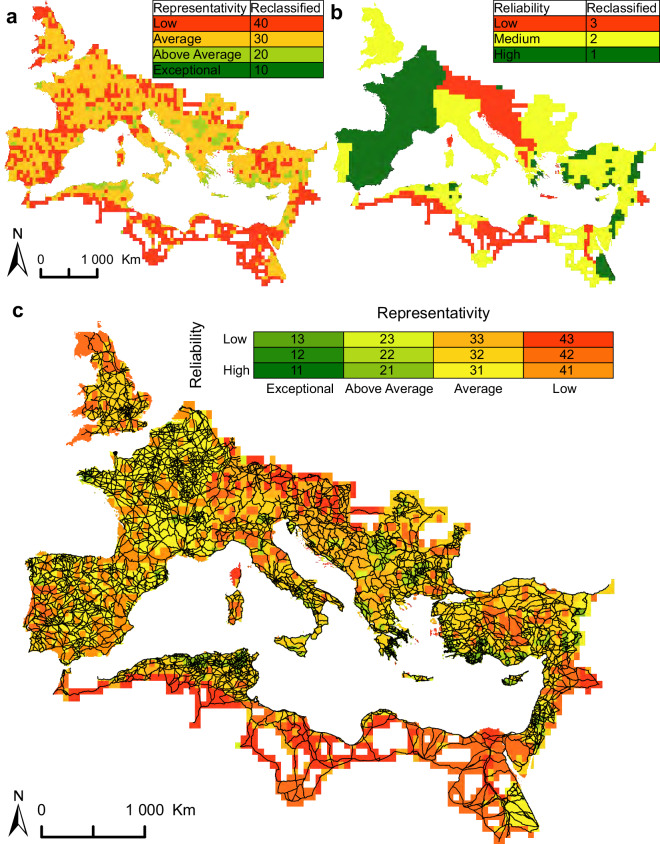
The confidence map is created by numerically reclassifying and summing the (**a**) representativity and (**b**) reliability maps. (**c**) Roman road data overlaid on the confidence map.

We evaluate and visualise this incompleteness and heterogeneity of the data by using the terminology and methodology proposed by Oštir *et al*.^316^ and Nuninger *et al*.^317^ to create a set of maps: road density (Fig. 5a) and number of vertices (Fig. 5b) are combined in a representativity map (Fig. 5c), which is in turn combined with source reliability (Fig. 6b), into a final confidence map (Fig. 6c). By establishing these maps as the basis for current knowledge about Roman roads, it will be possible to address some of these gaps through future projects that will further improve the digitised routes on our map through collaborative efforts.

### Units of analysis

The research area was divided into polygons measuring 0.5° × 0.5° (latitude and longitude). Polygons overlapping the ocean and seas were clipped to follow the coastlines. Polygons were only included in the confidence maps if they met two criteria: 1) intersecting with at least one road segment; 2) within the boundaries of the Roman Empire. This means polygons containing no road data in our dataset were excluded from the confidence analysis, including desert areas and small islands (e.g., Malta). The results for polygons at the borders of the analysis region (Fig. 5) suffer from edge effects: they receive lower values than would be expected, due either to the polygon that extends beyond the borders of the Empire not covered in this research (Lower Rhine, Upper and Middle Danube) or to small seacoast polygons with few road segments.

### Representativity map

The aim of the representativity map (Fig. 5c) is to visualise and quantify the spatial heterogeneity, resolution, and accuracy of the collected data. Representativity in the current context of the road dataset means two things: a) whether a region contains many roads that likely represent a complete road network (coverage), and b) whether roads are digitised in high detail (spatial resolution). To describe the dataset regarding coverage and resolution we use road density (m/km^2^, Fig. 5a) and vertices (number of vertices/km, Fig. 5b). Vertices represent edit points of each road segment in our dataset and in this way directly reflect the level of spatial resolution with which each road segment was digitised (roads digitised in higher resolution will have more vertices than roads digitised in lower resolution). The data were standardised within the polygons (units of analysis) in relation to the global (theoretical) mean value. In this way, we can compare the differences across regions to find areas where the data conforms to the average, is overrepresented, or is underrepresented compared to the global mean value. On the one hand it will be possible to identify areas of high road density and high resolution, which are therefore highly representative of the potentially complete Roman road network. On the other hand it will be possible to identify areas of low road density and low resolution, i.e., areas where the road network is underrepresented in the dataset. We employ these two metrics (road density and vertices) to analyse representativity for several reasons. Taking into account only road density would underrepresent areas where lower road density is expected (such as mountains, deserts, etc.). On the other hand, considering only vertices would overrepresent areas with complicated road profiles – such as mountains where roads tend to be more sinuous than on plains. By combining these two metrics it is possible to better capture overall representativity of the dataset and to mitigate effects caused by its heterogeneity.

#### Road density

The mean value used is the global road density computed as the fraction of total road length over the total area of the polygons.

Global road density = 299,171,310.141 m / 4,819,775 km^2^  = 62.07 m/km^2^

The road density for each polygon is then calculated and the mean expected value (global road density) is subtracted from the values in the polygons.

Difference in road density = Road density in a polygon – Global road density

The resulting difference values are then classified into four categories representing the difference of the local road density compared to the global road density calculated in multiples of the global road density 62.07 (Table 3). The cut-off values were adapted and modified from the original methodology used by Oštir and Nuninger^316,317^. A value of ‘0’ represents no difference from the global road density, so the value between 0–1 times the global road density are considered as ‘Average’ representativity. Below ‘0’ signifies lower representativity than the global road density. The values for ‘Above Average’ and ‘Exceptional’ represent a difference of 1–4 times, and more than 4 times higher than the difference from the global road density respectively.

**Table 3:** Representativity categories based on differences in road density.

Difference in road density	Representativity	Reclassified values
<0	Low (also missing values)	40
>= 0 <=62.07	Average	30
>62.07 <=248.37	Above Average	20
> 48.37	Exceptional	10

‘0’ represents no difference from the global road density; categories are binned by 1 and 4 multiples of the global road density 62.07.

#### Vertices

The values of vertices per road length are normalised in a similar fashion as with the road density, first a global value is calculated:

Global vertices per road length = 1,031,791 vertices / 299,171.31 km = 3.448 vertices/km

The values are aggregated for each polygon of analysis and the global value is subtracted from the value of each polygon. This difference is divided into four categories (Table 4), based on the same rule as was done previously with the road density categories above.

**Table 4:** Representativity categories based on differences in vertices/road length.

Difference in vertices/road length	Representativity	Reclassified values
<=0	Low (also missing values)	40
>0 <=3.447	Average	30
>3.447 <=13.788	Above Average	20
>13.788	Exceptional	10

‘0’ represents no difference from global vertices per road length; categories are binned by 1 and 4 multiples of the global vertices per road length 3.447.

#### Representativity map results

To compose the final representativity map, the reclassified values of both road density and vertex count (Tables 3 and 4) are added together and divided by two (both categories are given equal weight). The resulting number is rounded down to arrive at the final representativity value used in the representativity map (Fig. 5c, Table 5). E.g., if the road density value is ‘Average’ (30) and vertex count ‘Above Average’ (20), the resulting value is calculated as 30 + 20 = 50, 50/2 = 25, and 25 is rounded to 20 (‘Above Average’). We chose to round the values to the ‘better’ category in order to reflect that at least one of the used metrics is more representative for the description of the data in the affected unit of analysis.

**Table 5:** Representativity values used in the final representativity map.

Representativity	Reclassified value
Low (also missing values)	40
Average	30
Above Average	20
Exceptional	10

The representativity map (Figs. 5c; 6a) visualises and quantifies the spatial heterogeneity, resolution, and accuracy of the collected data^316,317^. Of the 38.93% of the study area that has low representativity, 20.63% concerns deserts and 6.48% mountainous areas. Future data collection efforts should focus in particular on the remaining 11.82% of low representativity in areas including Northern England, Cornwall, the Upper and Middle Danube, Tuscany and Marche in Italy, Corsica, Sardinia, and central Anatolia.

### Reliability map

The aim of the reliability map (Fig. 6b; Table 6) is to assess the quality of the sources used for the road data collection. This represents a value judgement about the sources used by the researchers responsible for the data collection on a region-by-region basis. Three categories of reliability are defined: high, medium, and low. High reliability represents areas with ample and detailed academic publications, often composed of large national projects (e.g., the *Carte archéologique de la Gaule* in France) or regional surveys and syntheses providing data in high-resolution spatial accuracy. Medium reliability is reserved for less detailed and lower-resolution sources, which in our case include some of the previous efforts mapping the Roman world (the *Barrington Atlas of the Greek and Roman World*, *Tabula Imperii Romani*, and *Tabula Imperii Byzantini*) whose data were re-digitised and upscaled using additional sources and our methodology described above. Low reliability represents regions with little coverage in past scholarship or with very low resolution of data and therefore low reliability of the digitised geometry of the roads. In other instances, it represents areas where our own digitisation efforts only used previous large-scale and low-resolution resources (the *Barrington Atlas*). The reliability categories are reclassified into a numeric code using the following key:

**Table 6:** Reliability categories.

Reliability	Reclassified value
Low	3
Medium	2
High	1

The high reliability areas in North Africa, the Near East, Asia Minor, and Greece represent some of the more recent detailed regional-focused research projects (e.g., the Desert Networks project in the Eastern Desert of Egypt). The high reliability of Spain is thanks to the previous efforts of the Mercator-e project (https://fabricadesites.fcsh.unl.pt/mercator-e/). The high reliability of France and the Benelux countries is thanks to research carried out by project collaborators (Philip Verhagen and Toon Bongers). The moderate reliability of Portugal, Britain, Italy and the eastern Balkans is due to employing lower resolution sources, mostly region-based overviews. Most low reliability desert areas represent regions with only ‘Hypothetical’ road segments, i.e., segments with very low reliability in the digitised geometry and outdated scholarship. The two large Mediterranean islands – Corsica and Crete – together with the western Balkans and the Upper and Middle Danube region are covered only using the low-resolution maps published in the *Barrington Atlas*.

### Confidence map

The representativity and reliability maps were subsequently combined to create the confidence map (Fig. 6c). It is meant as a tool to evaluate confidence and reliability in spatial analysis results that use the data^316,317^. The representativity and reliability maps are reclassified into numerical values, rasterized, and using map algebra added up to create a simple matrix of summed representativity and reliability.

The confidence map (Fig. 6c) reveals areas for which open digital data collection should be prioritised: categories 33, 42 and 43 (39.51%). Among the more significant missing data gaps in this category are several areas of northern Italy (Tuscany, Marche, and northern Po valley), the western Danube, western Balkans, and Corsica. Other areas in these categories concern deserts or suffer from edge effects (see Units of analysis). Curious is the case of high reliability and low representativity areas mainly in Western Europe (category 41, 7.73%) – some (especially upland) areas exhibit lower digitisation resolution, while in other areas the environmental factors influencing human settlement and movement potential might lower representativity (such as floodplains of the lower Rhine and Landes de Gascogne).

While developing the methodology, we consulted with area experts to better understand local historical and environmental conditions that shaped the Roman road network. This expert knowledge helped to improve our methodological workflow and our understanding of the Roman road network in its complexity. There is inherent bias in all archaeological and historical data, stemming from human and geomorphological transformation processes, accidents of survival, and research biases. Throughout this process we attempted to minimise the potential for errors and omissions that would negatively impact the resulting dataset. The resulting dataset reflects these biases as shown on the confidence map, whilst representing the most comprehensive open digital dataset of Roman roads.

## Usage Notes

**Fig. 7:**
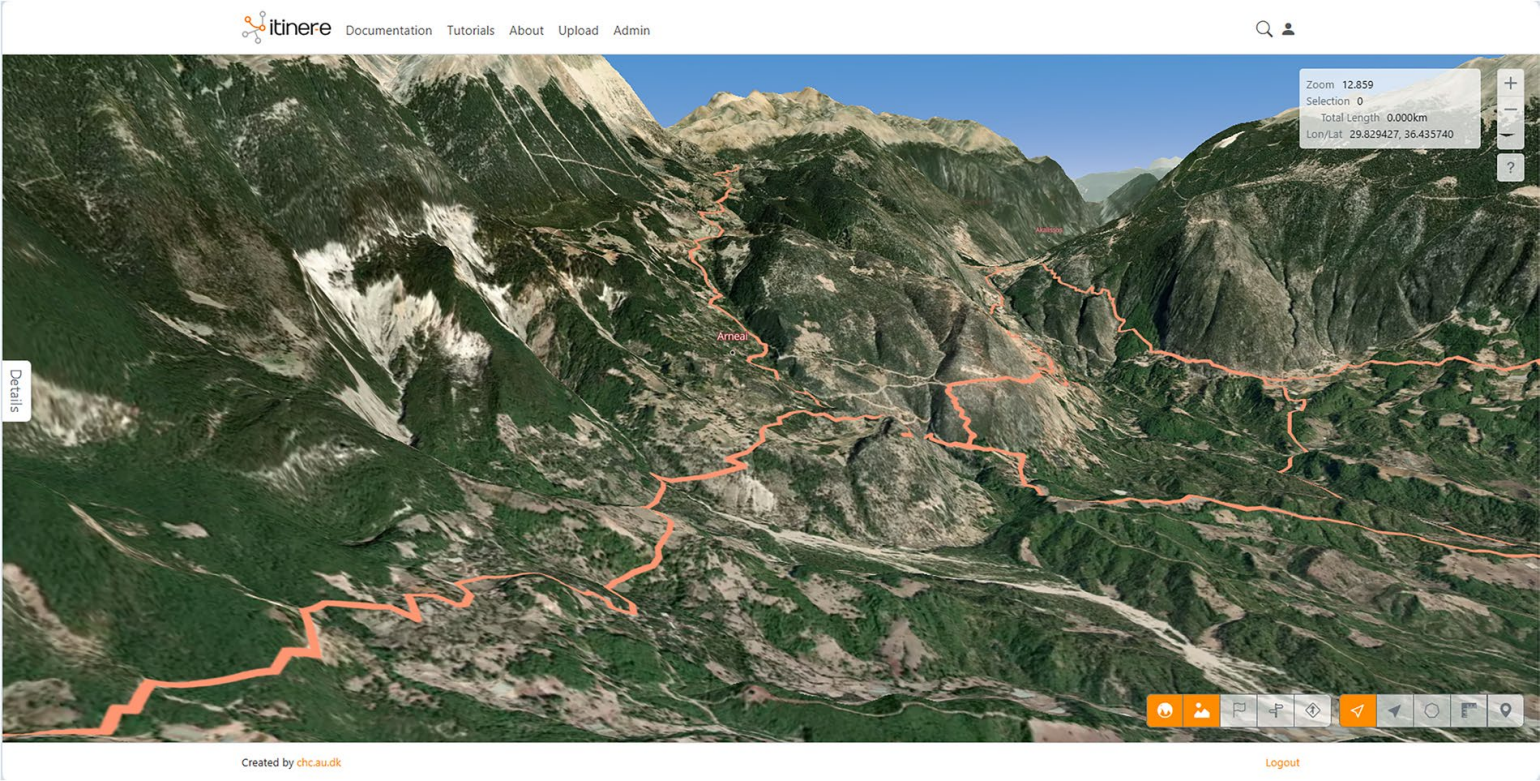
Screenshot of Itiner-e.org, a linked open data gazetteer for managing, querying, editing and expanding data about ancient roads, including a routing tool for recreating ancient journeys over roads and an immersive 3D landscape representation.

Itiner-e is valuable for future research on ancient mobility and trade, and the long-term development of transport infrastructure in Europe, the Near East and North Africa. Its spatial resolution means it can be used for both regional and Empire-wide studies. Our confidence map can be used to support future data collection work that can focus on areas of particularly low reliability and representativity to continue to fill in the gaps. Such focused work can expect particular growth in the knowledge about the existence and location of secondary roads. We also present an online platform (https://itiner-e.org) that allows Itiner-e to grow, and to address the incompleteness and heterogeneity revealed by our confidence maps (Fig. 7). The Itiner-e platform allows a scholarly community to add new road data as it becomes available, or to recommend corrections and alternative interpretations of existing road data.

## Data Availability

The dataset is available in an open Zenodo^314^ repository (10.5281/zenodo.17122148) and with a canonical citation on the Itiner-e platform (https://itiner-e.org). The Zenodo repository contains the data stored in shapefile format (itinere_roads.shp and associated files), as a geopackage (itinere_roads.gpkg), and as a geojson database (itinere_roads.geojson). In addition, it contains a data field description file (Data field description.docx) and bibliography file (Itiner-e bibliography.bib).

## Code availability

No customised code was produced to create or analyse this dataset. Specifically, the data was manually digitised with a target spatial resolution of 5–200 m on a GIS platform and stored as a single data record in a vector layer with an associated attribute table. The dataset is available on Zenodo at: 10.5281/zenodo.17122148.
